# Supplementary material for: Role and mechanism of NCAPD3 in promoting malignant behaviors in gastric cancer
Source: Front Pharmacol. 2024 Apr 22;15:1341039. doi: 10.3389/fphar.2024.1341039 (PMC11070777; doi:10.3389/fphar.2024.1341039)
Supplement: Supplementary file 11 [file DataSheet2.ZIP › GSEA/immunologic/my_analysis.Gsea.1599462003817/GSE29618_PDC_VS_MDC_DAY7_FLU_VACCINE_DN.html]

Details for gene set GSE29618\_PDC\_VS\_MDC\_DAY7\_FLU\_VACCINE\_DN[GSEA]

|  || Dataset | filtered\_dataset.sample\_info.cls#WT\_versus\_NCAPD3\_MUT |
| Phenotype | sample\_info.cls#WT\_versus\_NCAPD3\_MUT |
| Upregulated in class | NCAPD3\_MUT |
| GeneSet | GSE29618\_PDC\_VS\_MDC\_DAY7\_FLU\_VACCINE\_DN |
| Enrichment Score (ES) | -0.56056136 |
| Normalized Enrichment Score (NES) | -2.4681804 |
| Nominal p-value | 0.0 |
| FDR q-value | 0.014155348 |
| FWER p-Value | 0.132 |
Table: GSEA Results Summary

  

Fig 1: Enrichment plot: GSE29618\_PDC\_VS\_MDC\_DAY7\_FLU\_VACCINE\_DN      
 Profile of the Running ES Score & Positions of GeneSet Members on the Rank Ordered List

  

| SYMBOL | TITLE | RANK IN GENE LIST | RANK METRIC SCORE | RUNNING ES | CORE ENRICHMENT || 1 | 1979 | EIF4EBP2 | 532 | 0.456 | -0.3330 | No |
| 2 | 6303 | SAT1 | 850 | -0.233 | -0.5356 | Yes |
| 3 | 1051 | CEBPB | 862 | -0.274 | -0.5141 | Yes |
| 4 | 6515 | SLC2A3 | 873 | -0.283 | -0.4910 | Yes |
| 5 | 10797 | MTHFD2 | 911 | -0.338 | -0.4814 | Yes |
| 6 | 301 | ANXA1 | 914 | -0.342 | -0.4461 | Yes |
| 7 | 6397 | SEC14L1 | 941 | -0.364 | -0.4258 | Yes |
| 8 | 2037 | EPB41L2 | 943 | -0.365 | -0.3873 | Yes |
| 9 | 7277 | TUBA4A | 997 | -0.404 | -0.3821 | Yes |
| 10 | 3516 | RBPJ | 1021 | -0.419 | -0.3537 | Yes |
| 11 | 80005 | DOCK5 | 1045 | -0.441 | -0.3229 | Yes |
| 12 | 7204 | TRIO | 1120 | -0.494 | -0.3231 | Yes |
| 13 | 604 | BCL6 | 1136 | -0.504 | -0.2798 | Yes |
| 14 | 50856 | CLEC4A | 1249 | -0.630 | -0.2926 | Yes |
| 15 | 202 | AIM1 | 1290 | -0.681 | -0.2483 | Yes |
| 16 | 51703 | ACSL5 | 1353 | -0.800 | -0.2071 | Yes |
| 17 | 7462 | LAT2 | 1399 | -1.138 | -0.1174 | Yes |
| 18 | 1475 | CSTA | 1400 | -1.162 | 0.0072 | Yes |
Table: GSEA details [plain text format]

  

Fig 2: GSE29618\_PDC\_VS\_MDC\_DAY7\_FLU\_VACCINE\_DN      
 Blue-Pink O' Gram in the Space of the Analyzed GeneSet

  

Fig 3: GSE29618\_PDC\_VS\_MDC\_DAY7\_FLU\_VACCINE\_DN: Random ES distribution      
 Gene set null distribution of ES for **GSE29618\_PDC\_VS\_MDC\_DAY7\_FLU\_VACCINE\_DN**
